# Deep-learning generated B-line score mirrors clinical progression of disease for patients with heart failure

**DOI:** 10.1186/s13089-024-00391-4

**Published:** 2024-09-16

**Authors:** Cristiana Baloescu, Alvin Chen, Alexander Varasteh, Jane Hall, Grzegorz Toporek, Shubham Patil, Robert L. McNamara, Balasundar Raju, Christopher L. Moore

**Affiliations:** 1 Department of Emergency Medicine, Yale University School of Medicine, 464 Congress Avenue, Suite 260, New Haven, Connecticut 06519 USA; 2 Philips Research Americas, 222 Jacobs Street, Cambridge, MA 02141 USA; 3 Present Address: Department of Emergency Medicine, Washington University School of Medicine, 660 S. Euclid Avenue, St. Louis, MO 63110 USA; 4 Department of Emergency Medicine, University of Washington, Seattle, WA USA; 5 Present Address: Inari Medical, One Kendall Square, Building 600/700, Suite 7-501, Cambridge, MA 02139 USA; 6 Division of Cardiology, Department of Internal Medicine, Yale University School of Medicine, PO Box 208017, New Haven, CT 06520 USA

**Keywords:** Point-of-care ultrasound, Lung ultrasound, B-lines, Heart failure, Artificial intelligence

## Abstract

**Background:**

Ultrasound can detect fluid in the alveolar and interstitial spaces of the lung using the presence of artifacts known as B-lines. The aim of this study was to determine whether a deep learning algorithm generated B-line severity score correlated with pulmonary congestion and disease severity based on clinical assessment (as identified by composite congestion score and Rothman index) and to evaluate changes in the score with treatment. Patients suspected of congestive heart failure underwent daily ultrasonography. Eight lung zones (right and left anterior/lateral and superior/inferior) were scanned using a tablet ultrasound system with a phased-array probe. Mixed effects modeling explored the association between average B-line score and the composite congestion score, and average B-line score and Rothman index, respectively. Covariates tested included patient and exam level data (sex, age, presence of selected comorbidities, baseline sodium and hemoglobin, creatinine, vital signs, oxygen delivery amount and delivery method, diuretic dose).

**Results:**

Analysis included 110 unique subjects (3379 clips). B-line severity score was significantly associated with the composite congestion score, with a coefficient of 0.7 (95% CI 0.1–1.2 *p* = 0.02), but was not significantly associated with the Rothman index.

**Conclusions:**

Use of this technology may allow clinicians with limited ultrasound experience to determine an objective measure of B-line burden.

## Background

Thoracic ultrasound has emerged as a vital tool in assessing hospitalized patients experiencing shortness of breath, particularly in the initial evaluation within the Emergency Department (ED) [1–4]. Lung ultrasound, in particular, can detect alveolar interstitial syndrome (AIS), indicating the presence of fluid in the lung alveolar and interstitial spaces. AIS is characterized by the appearance of “B-lines”, ring-down artifactsextending from the pleural line to the bottom of the screen thatmove with respiration [5–7].

B-lines can be seen in several different conditions such as pulmonary edema in acute heart failure (HF), pneumonia, pulmonary embolus, end stage renal disease with volume overload, acute respiratory distress syndrome, and COVID-19 [4, 7–15]. The quantity and morphology of B-lines correlate with the amount of interstitial fluid present and loss of lung aeration [16, 17]. AIS severity can be a prognostic factor in heart failure and renal failure [18–20]. B-line severity in critically ill patients has been found to be predictive of mortality, length of stay and time on the ventilator [21]. However, B-line identification and quantification can vary based on user experience [22–24].

Leveraging machine learning, specifically artificial intelligence (AI) based quantification methods, may improve diagnosis when properly employed [25–27]. AI algorithms, especially those created using deep neural networks (deep learning), are increasingly utilized in medical research to generate substantial amounts of data for large scale projects [21, 28]. Employing AI to rate B-line severity allows for rapid processing of sizable datasets and increases feasibility of large-scale research studies. Automated interpretation may also allow more reproducible measures of B-line severity and could potentially be obtained by users with less experience in ultrasound (e.g. a nurse monitoring progress of a patient with pulmonary edema) and may enhance ultrasound utility in low resource settings. Employing AI in healthcare settings can refine cardiac disease evaluation, enhancing accuracy, efficiency, and personalized care, ultimately improving patient outcomes and resource allocation [29, 30].

While some studies have shown dynamic changes in B-line severity with response to treatment, fewer have examined B-line severity evolution over the treatment course and correlation with symptomatology or severity indicators, particularly in inpatient settings [18, 19, 31]. Changes in B-line severity are anticipated to parallel the clinical trajectory, potentially serving as an additional indicator of inpatient treatment advancement and efficacy. Illness severity categorization can offer valuable insights across various clinical scenarios and guide medical decision-making. Illness severity scores typically gauge the extent of illness and mirror the intricacies of the disease progression but are also used for predictive and comparative outcome assessment, resource allocation, and care process evaluation [32]. Examples include Modified Early Warning Score, Acute Physiology and Chronic Health Evaluation, Rothman index, and others [33–35]. At present, there is no established standardized score for evaluating severity during admissions for heart failure, making the assessment of clinical severity complex [36]. While congestion is primarily diagnosed clinically, a composite congestion score has been outlined to track the advancement of congestive signs and symptoms in response to standard therapy in heart failure [37]. Less is known about how B-line severity correlates with scores of illness severity categorization and if B-line severity may augment this assessment.

The aim of this study was to determine the association between a deep-learning generated B-Line severity score and the degree of pulmonary congestion severity based on clinical assessment without ultrasound (via composite congestion score) and overall illness severity (based on Rothman index) in patients with suspicion of heart failure induced pulmonary edema, and its changes in response to inpatient treatment.

## Methods

### Study design

This was a prospective, observational study conducted at a large academic medical center between July 2018 and May 2019. Subjects included adult English-speaking patients who presented to the Emergency Department (ED) with dyspnea and/or hypoxia, were triaged to the high acuity section of the ED, displayed B-lines on an initial screening ultrasound, and had a diagnosis of heart failure or pulmonary edema upon admission or discharge. Additionally, subjects admitted to the heart failure floor with a diagnosis of pulmonary edema or heart failure were enrolled as a supplemental cohort. The study was approved by local institutional review board. All patients meeting inclusion criteria were approached for enrollment consecutively during defined periods and invited to participate.

### Subject and exam-level data collection

The study collected subject demographic information such as age, gender, race, and ethnicity, alongside various clinical parameters recorded at the time of each study ultrasound. These included vital signs, oxygen delivery method, fraction of inspired oxygen (FiO2), patient position (bed angle), recent laboratory results (troponin I, troponin T, N-terminal pro b-type natriuretic peptide), radiological imaging results (chest X-ray, lung computer tomography, transthoracic echocardiograms), and medications relevant to heart failure and pulmonary edema (type and dosage of diuretics, type and dosage of vasodilators).

Additionally, clinical data such as fluid intake and output for the 24 h before the ultrasound were documented. Intravenous and oral loop diuretic dosages were converted to intravenous furosemide equivalents [38]. Percent FiO2 was estimated by converting the flow rate into an approximate percentage. For example, two liters of oxygen via nasal cannula was considered 24% FiO2, increasing by 4% per liter up to six liters. Non-rebreather masks or high-flow nasal cannulas were approximated as 90% FiO2. Charted FiO2 was recorded for ventilated patients. Subjects were classified as having a diagnosis of heart failure or pulmonary edema based on the presence of either diagnosis in their recorded admission or discharge diagnoses. In cases where this was unclear (nonspecific diagnoses such as shortness of breath, dyspnea, acute respiratory failure, etc.), electronic medical records were reviewed by a blinded author to determine the diagnosis, independent of B-line severity.A Composite Congestion Score (CCS) was calculated for each subject at the time of each research ultrasound. Dyspnea, fatigue, orthopnea, jugular venous distension, rales, and pedal edema were evaluated and documented prospectively by the research assistant using a standardized 4-point scale (Table 1) [37]. The points were then aggregated to determine the final CCS, a metric previously outlined in the EVEREST trial [37].

**Table 1:** Composite congestion score (scale for investigator-assessed signs and symptoms of congestion) [37]

Signs/symptoms	0	1	2	3
Dyspnea	None	Seldom	Frequent	Continuous
Orthopnoea	None	Seldom	Frequent	Continuous
Fatigue	None	Seldom	Frequent	Continuous
JVD (cm H_2_O)	≤ 6	6–9	10–15	≥ 15
Rales	None	Bases	To < 50%	To > 50%
Edema	Absent/trace	Slight	Moderate	Marked

JVD, jugular venous distension

Additionally, the Rothman index was also collected as a potential indicator of overall illness severity [33]. The study recorded admission and discharge diagnoses of the subjects. Readmission events and survival status were monitored for six months from the initial enrollment date. Lung ultrasound clips, clinical parameters, Rothman index and CCS were collected daily until either discharge or the 10th day of hospitalization. Data collection was timed as close to 24-hour intervals as possible.

### Lung ultrasound examinations

**Fig. 1:**
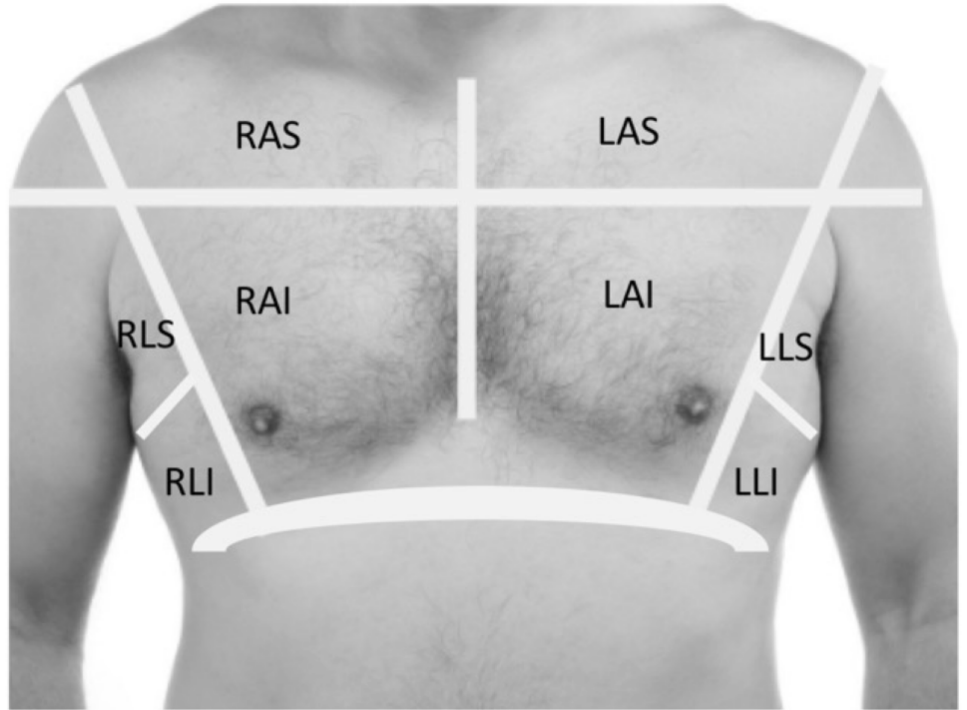
Location of ultrasound interrogation of thorax for B-line presence and quantity: right anterior superior (RAS), right anterior inferior (RAI), right lateral superior (RLS), right lateral inferior (RLI), left anterior superior (LAS), left anterior inferior (LAI), left lateral superior (LLS) and left lateral inferior (LLI)

Hospitalized subjects underwent daily 8-zone lung ultrasound examinations during their inpatient stay. Lung ultrasound exams were performed by a trained research assistant using a Philips Lumify S4-1 phased array transducer with depth set to 15 cm. Recordings of three-second clips were obtained from 8 distinct lung zones (right and left anterior superior, anterior inferior, lateral superior, and lateral inferior, Fig. 1) while the patient assumed a comfortable position, typically at approximately a 45-degree angle. For subjects enrolled in the Emergency Department (ED), ultrasound scans were repeated once on the day of enrollment, with a time gap of 2 to 5 h if the patient remained in the ED. If significant events like positive pressure ventilation, nitroglycerin drip, or diuretic administration occurred during the ED stay, and the patient was ready for transfer to an inpatient floor within 2 h, the scan was repeated sooner, 1–2 h later i. Clips were de-identified using ClipDeidentifier (www.ultrasoundoftheweek.com) for MP4 format, and DICOM Cleaner (PixelMed Publishing™) for DICOM format.

### Deep-learning generated B-line score

B-line severity in each video loop was assessed using a modified version of a previously published deep learning algorithm, which rates the severity of B-lines on a scale from 0 to 4 [28, 39].

**Fig. 2:**
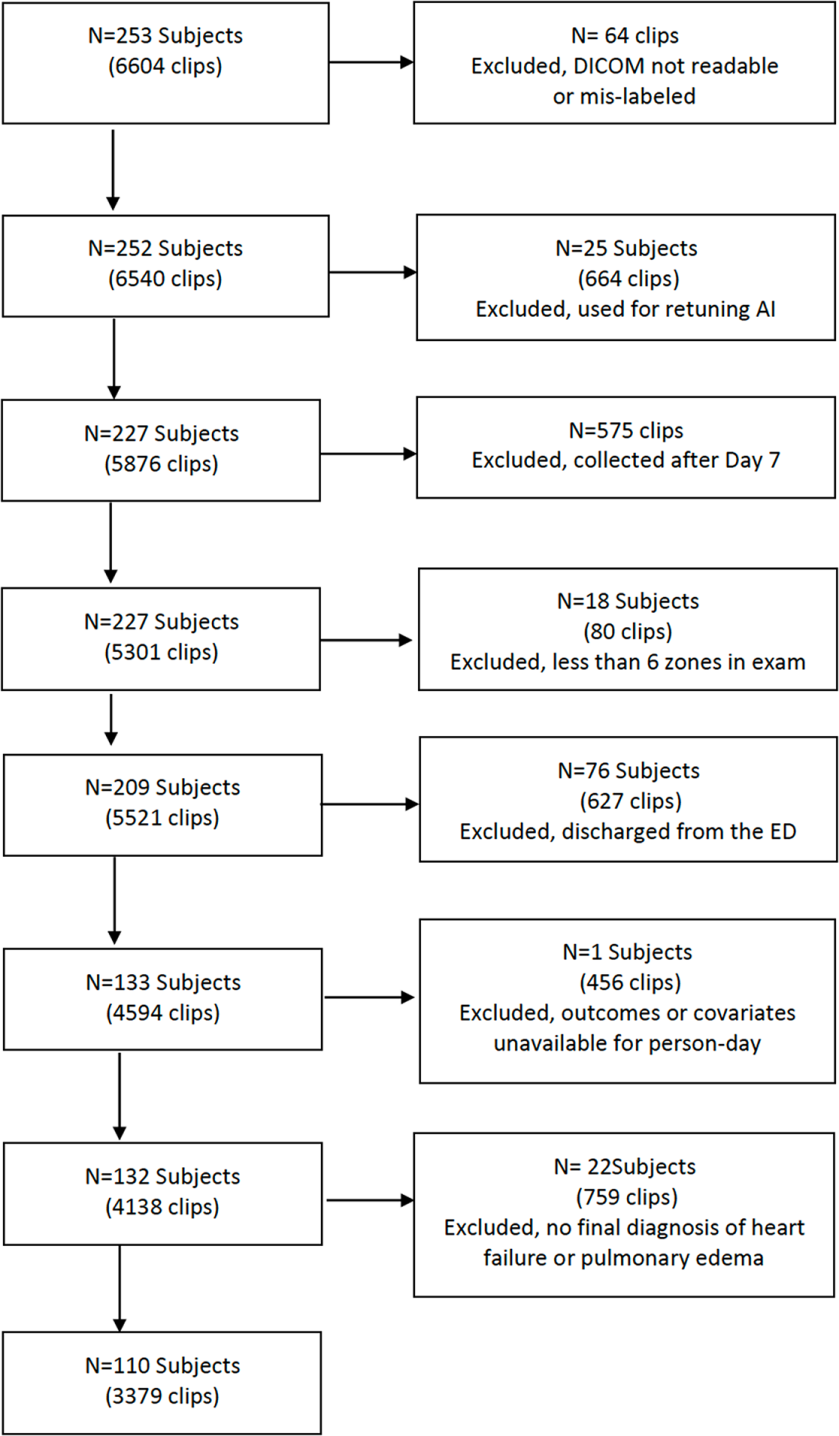
Flow diagram of enrollment and clips utilized in the study

A total of 838 exams were conducted on 253 subjects, resulting in a dataset of 6,604 clips (video loops). Each video loop contained approximately 90 frames (30 frames per second). Clips that were unreadable DICOM files, mislabeled data, and those used for algorithm retuning (as described in the prior publication utilizing the same large dataset), were excluded [28]. Further excluded were exams with clips from less than 6 zones, exams from subjects discharged after ED evaluation, those lacking outcomes or model covariates available for person-day, and subjects without a diagnosis of heart failure or pulmonary edema (Fig. 2). Final dataset contained 3379 clips from 110 subjects.

### Statistical analyses

A multi-level mixed effects modeling approach was employed to explore the association between B-lines and clinical severity over time. This modeling technique allows for grouping patients to consider individual variations when dealing with longitudinal data that includes multiple measurements per patient [40]. The study evaluated the associations between these covariates and the deep-learning generated B-line score with either CCS or Rothman index. Candidate covariates encompassed demographic and clinical characteristics selected via clinician gestalt, excluding those with over 30% missing data (N-terminal pro-brain natriuretic peptide and intake-output). Reverse stepwise selection was used for model creation and eliminated the least significantly associated covariates with a stopping threshold of *p* = 0.05. Variable inflation factor was calculated to rule out for multi-collinearity among the final model covariates. No data imputation or replacement for missing data was performed. Days with missing data, either clinical or all 8-zone scores, were excluded from the analyses. All statistical analyses were performed using Stata (v.15.1, College Station, TX).

## Results

**Fig. 3:**
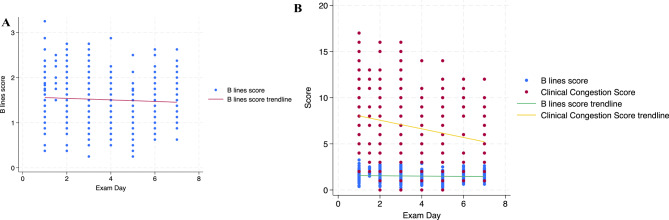
**a** Eight-zone average daily B-line score trendline over hospitalization days. **b** B-line score and Composite Congestion score trendlines

The primary study cohort, comprising 110 subjects (423 patient-days, 3379 clips), was analyzed to explore the association between clinical severity (represented by CCS and Rothman index) and deep-learning generated B-line severity The average age of subjects was 72 years old (±13 years), and 45% were female (Table 2). The average 8-zone B-line score over hospitalization duration was down trending, as displayed in Fig. 3a.

**Table 2:** Patient characteristics

*N* (%) or Mean (SD)	Cohort of patients with heart failure/pulmonary edema *N* = 110
Demographics/Enrollment	
Age	71.8 (12.9)
Female	49 (44.5)
Location of enrollment (ED)	64 (58.2)
Vital Signs	
Heart Rate	80.1 (17.6)
Systolic Blood Pressure	126.8 (22.1)
Diastolic Blood Pressure	70.1 (15.1)
Respiratory Rate	19.2 (2.4)
O2 Delivery Type	
Nasal cannula	55 (50.0)
Room air	53 (48.1)
Other	2 (1.8)
FiO_2_	27.9 (12.1)
Baseline Lab studies	
Sodium	138.9 (4.5)
Hemoglobin	11.4 (2.4)
NT-ProBNP	9028.1 (13894.6)
Creatinine	1.7 (1.1)
Diuretic Type	
Combination	15 (13.6)
Loop Only	78 (70.9)
None	16 (14.5)
Diuretic Dose	99.5 (120.7)
Findings/Outcomes	
Baseline Composite Congestion Score	8.7 (3.2)
Baseline Rothman Score	61.1 (15.4)
Readmission (non-procedural) within 90 days	44 (40%)

A weak unadjusted association was observed between composite congestion score and deep-learning generated B-line severity (Fig. 3b). Adjustment via the mixed effects regression model demonstrated a statistically significant association between the deep-learning generated B-line score and CCS, with a coefficient 0.7 (95% CI 0.1 to 1.2, *p* = 0.02, Table 3). The other covariates included in the final model were total loop diuretic dose on day of exam, respiratory rate, baseline hemoglobin and sodium, FiO2, and lack of supplemental oxygen delivery (room air oxygen delivery type).

**Table 3:** Mixed effects modeling investigating association between composite congestion score and B-line severity

Composite Congestion Score	Coefficient	95% CI
B-line severity AI Rating	0.7	0.1 to 1.2
Exam Day (1–7)	-0.6	-0.7 to -0.5
Total loop diuretic dose	0.001	-0.001 to 0.002
Respiratory Rate	0.1	-0.02 to 0.16
Baseline Hemoglobin	-0.3	-0.5 to -0.1
O2 delivery type: room air	-2.4	-3.1 to -1.7
FiO2	-0.02	-0.06 to 0.02
Baseline Sodium	-0.1	-0.2 to -0.03

There was no significant association between Rothman index and deep-learning generated B-line severity in either unadjusted or adjusted analyses (Table 4).

**Table 4:** Mixed effects modeling investigating association between Rothman Index and B-line severity

Rothman Index	Coefficient	95% CI
B-line severity AI Rating	-0.5	-2.5 to 1.6
Exam Day	-0.2	-0.7 to 0.3
Comorbidity – Afib	-3.2	-7.3 to 0.9
Comorbidity – COPD	-3.8	-8.7 to 1.1
Age	-0.1	-10.9 to -2.3
Female (vs. Male)	-6.6	-0.2 to 2.0
Total Loop Diuretic Dose	-0.004	-0.01 to 0.003
Diastolic blood pressure	0.09	0.004 to 0.17
Respiratory Rate	-0.8	-1.2 to -0.5
Baseline sodium	0.5	0.02 to 1.0
Baseline hemoglobin	1.4	0.5 to 2.2
FiO2	-0.1	-0.2 to 0.03

## Discussion

This study contributes to the body of evidence indicating that B-lines are dynamic artifacts, and that B-line severity decreases over time as congestion symptoms decrease. We have demonstrated that an AI scoring system developed using deep learning correlates with changes in clinical severity of pulmonary congestion. AI generated B-line severity in our study was significantly associated with symptom burden as measured by CCS, though not associated with the Rothman index.

The decision to use CCS was informed by a study by Ambrosy et al., which investigated the progression of congestive signs and symptoms in response to standard therapy in a large contemporary cohort of patients hospitalized for worsening heart failure with reduced ejection fraction [37]. The study showed significant improvement in CCS distribution from baseline to discharge, with a simultaneous reduction in CCS correlating with sustained body weight loss [37]. Patients with higher CCS were also more frequently categorized as New York Heart Association functional class IV [37]. These findings collectively support CCS as a marker of heart failure-induced pulmonary congestion.

Although the link between CCS and B-line severity displayed statistical significance, the estimate’s confidence interval is broad, suggesting a potentially weak clinical association. This may be because of some existing mismatch between congestion and B-line severity [41, 42]. B-lines have been found to persist at discharge despite lack of symptoms and clinical exam improvement: approximately 40% of patients with no rales show subclinical congestion with five or more B-lines on ultrasound at hospital discharge [43]. Additionally, in patients with renal failure, B-lines are found on ultrasound despite lack of pulmonary symptoms [20].

Another potential reason for the lack of strength of the association between CCS and B-line severity is that our study does not compare B-line severity with a fully objective assessment of clinical severity, as the CCS is partly based on symptom burden. At present, there is no established standardized and objective method for evaluating congestion in heart failure, making the assessment of clinical severity complex [36]. While chart review could have been used to assess clinical severity, conducting a daily severity review for the entire study patient population would have been highly resource-intensive for the study, and it is likely that retrospective capture of variables would yield incomplete and potentially inaccurate results.

The Rothman Index (RI, PeraHealth, Inc. Charlotte, NC, USA), an illness severity predictive model which uses continuous measurements of patient data from 26 non-static variables to measure physiologic acuity, was incorporated as a more objective comparator [33, 44]. This score is derived from vital signs, nursing assessments, laboratory findings, and cardiac rhythm, and is computed by assessing deviations from standard values, with a maximum score of 100 representing conformity to standard values. A decline in score corresponds to a deterioration in patient health. Designed to be applicable across patients regardless of diagnosis, procedure, or setting, the Rothman Index aims to offer healthcare providers a measurable, ongoing assessment of a patient’s clinical condition automatically generated by the electronic medical record [33]. However, the Rothman index does consider nursing reports, which can be subjective. Our study found no significant association between B-line severity and the Rothman Index. One would expect an inverse relationship if patients were improving, but the lack of association may not be entirely surprising since the Rothman Index is designed to predict patient deterioration, not illness severity per se [33]. Unlike other early warning systems like National Early Warning Score, the Rothman Index relies on a larger number of data inputs, but these data points are given equal weight, potentially causing fluctuations in one variable to offset changes in another [33, 45]. Noise that may be introduced by additional data inputs would cancel out signal from the most critical variables. One rationale for selecting the Rothman index compared to other measures was its widespread utilization as a clinical severity metric within the medical center where the study was conducted, thus ensuring its availability for all patients. Such a score would be readily available to entire medical team easily by review of electronic medical record.

While we attempted to use available tools such as the CCS and Rothman Index, it should be understood that there is really no agreed upon gold standard, particularly one that is readily obtainable, to objectively quantify pulmonary congestion. It is conceivable that an ultrasound measure of pulmonary congestion, particularly one that is more objectively determined using AI, may ultimately outperform these existing imperfect and subjective measures.

Another limitation of the study was inclusion criteria of patients with undifferentiated dyspnea “suspected” of heart failure, alongside an admitted heart failure cohort, which resulted in some diagnostic variability. Fisease processes other than CHF and pulmonary edema cause B-lines. Although most subjects ultimately received a discharge diagnosis of heart failure and pulmonary edema (pathologies associated with B-lines expected to improve over time), this may not have been true of patients who did not have CHF, or exhibited dual-diagnoses. For instance, a patient with lung cancer would be expected to have persistent lung findings despite their clinical course during a hospitalization, potentially biasing the results. To address this, we specifically enrolled patients with suspicion of heart failure and focused on evaluating the cohort with confirmed heart failure/pulmonary edema diagnoses to minimize the impact of this limitation in our analysis, but future studies could have stricter enrollment criteria and exclusion of other diagnoses causing fluid overload.

In patients with hypervolemia due to acute decompensated heart failure, lung ultrasound has improved sensitivity and specificity compared to chest x-ray (CXR) for pulmonary edema and pleural effusions [46–48]. In some settings, daily CXRs are common for monitoring, which can involve logistical challenges such as patient transport, costs, delays in interpretation, and radiation exposure [49]. In our study, B-line severity showed an independent association regardless of oxygenation needs and radiology results. Lung ultrasound offers a promising alternative to CXR as it is radiation sparing and can be conducted promptly at the bedside, potentially by a variety of staff members with the assistance of AI.

Using a deep-learning generated B-line score, we were able to use multi-level mixed effects modeling to test association of over twenty candidate covariates with CCS on this large dataset. Manual expert B-line scoring of > 3000 clips would have been impractical. In addition to AI assisted research dataset processing, AI-assisted lung ultrasound for real-time B-line severity assessment during hospitalization or at discharge potentially holds clinical benefits. Presence of B-lines at hospital discharge of patients with heart failure indicates a five-fold increased risk for readmission or death, and presence of B-lines predicts a four-fold risk for hospitalization or death for ambulatory chronic heart failure patients [19, 50]. B-lines outperform ejection fraction as predictors for death, myocardial infarction, and heart failure progression [51, 52]. This important predictive data maymerit a different follow-up approach than similar patients without significant B-line burden and would be important information to have during a hospital admission [53]. Tracking B-line severity during hospitalization could be especially valuable for critically ill populations or those unable to communicate their symptoms. AI aids non-expert clinicians in obtaining and interpreting lung ultrasound data, and AI-based risk prediction models integrating clinical and imaging variables offer personalized assessments for heart failure management, supporting treatment decision-making [30, 54].

## Conclusions

Our AI scoring system for B-line severity, generated via deep learning algorithm interpretation of lung ultrasound, was significantly associated with the composite congestion score. Use of this technology may allow clinicians with limited ultrasound experience to determine an objective measure of B-line burden. Further prospective testing of automated B-line assessment into diagnosis, prognosis, and therapy is warranted.

## Data Availability

The datasets used and/or analyzed during the current study are available from the corresponding author on reasonable request.

## Abbreviations

AI: Artificial Intelligence
CCS: Composite Congestion Score
FiO2: Fraction of inspired oxygen
